# Late presentation of arrhythmogenic right ventricular cardiomyopathy in an octogenarian associated with a pathogenic variant in the plakophilin 2 gene: a case report

**DOI:** 10.1186/s12872-019-1018-2

**Published:** 2019-02-19

**Authors:** Yusuke Adachi, Takekuni Hayashi, Takeshi Mitsuhashi, Kenichi Sakakura, Yoko Yamada, Yuko Wada, Minoru Horie, Shin-ichi Momomura, Hideo Fujita

**Affiliations:** 1 Division of Cardiovascular Medicine, Saitama Medical Center, Jichi Medical University, 1-847 Amanuma, Omiya, Saitama, 330-8503 Japan; 2 Department of Cardiovascular and Respiratory Medicine, Shiga University of Medical Science, Shiga, Japan

**Keywords:** Arrhythmogenic right ventricular cardiomyopathy, Octogenarian, Elderly, Plakophilin 2

## Abstract

**Background:**

Arrhythmogenic right ventricular cardiomyopathy (ARVC) is an inherited myocardial disease characterized by fibrofatty replacement and ventricular arrhythmias. ARVC is believed to be a disease of the young, with most cases being diagnosed before the age of 40 years. We report here a case of newly diagnosed ARVC in an octogenarian associated with a pathogenic variant in the plakophilin 2 gene (*PKP2*).

**Case presentation:**

An 80-year-old Japanese man was referred for sustained ventricular tachycardia. His baseline electrocardiogram showed negative T waves in V1–V4. Right ventriculography showed right ventricular aneurysm. Because this case met three major criteria, ARVC was diagnosed. He was successfully treated with radiofrequency ablation and oral amiodarone. Genetic analysis identified an insertion mutation in exon 8 of *PKP2* (1725_1728dupGATG), which caused a frameshift and premature termination of translation (R577DfsX5).

**Conclusions:**

To the best of our knowledge, this is the first report of newly diagnosed ARVC in an octogenarian associated with a loss-of-function *PKP2* pathogenic variant. Although the late clinical presentation of ARVC is rare, it should be included in the differential diagnosis when treating older patients with ventricular tachyarrhythmias.

**Electronic supplementary material:**

The online version of this article (10.1186/s12872-019-1018-2) contains supplementary material, which is available to authorized users.

## Background

Arrhythmogenic right ventricular cardiomyopathy (ARVC) is an inherited myocardial disease characterized by fibrofatty replacement and ventricular arrhythmias [1]. ARVC is believed to be a disease of the young, with most cases being diagnosed before the age of 40 years [2]. We report here a case of newly diagnosed ARVC in an octogenarian associated with a pathogenic variant in the plakophilin 2 gene (*PKP2*).

## Case presentation

**Fig. 1:**
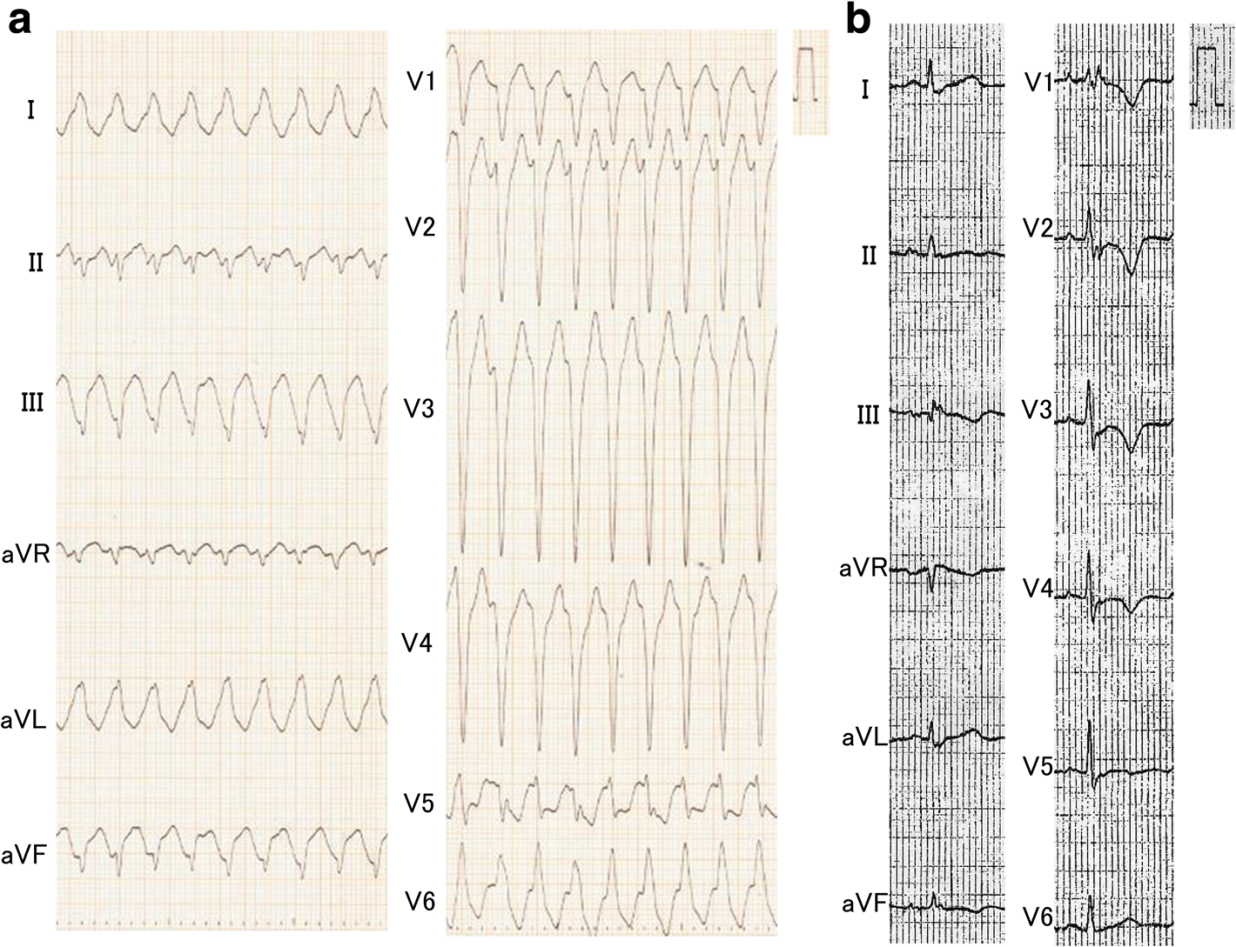
**a** A 12-lead ECG showing ventricular tachycardia. **b** Baseline ECG showing negative T waves in V1–V4

**Fig. 2:**
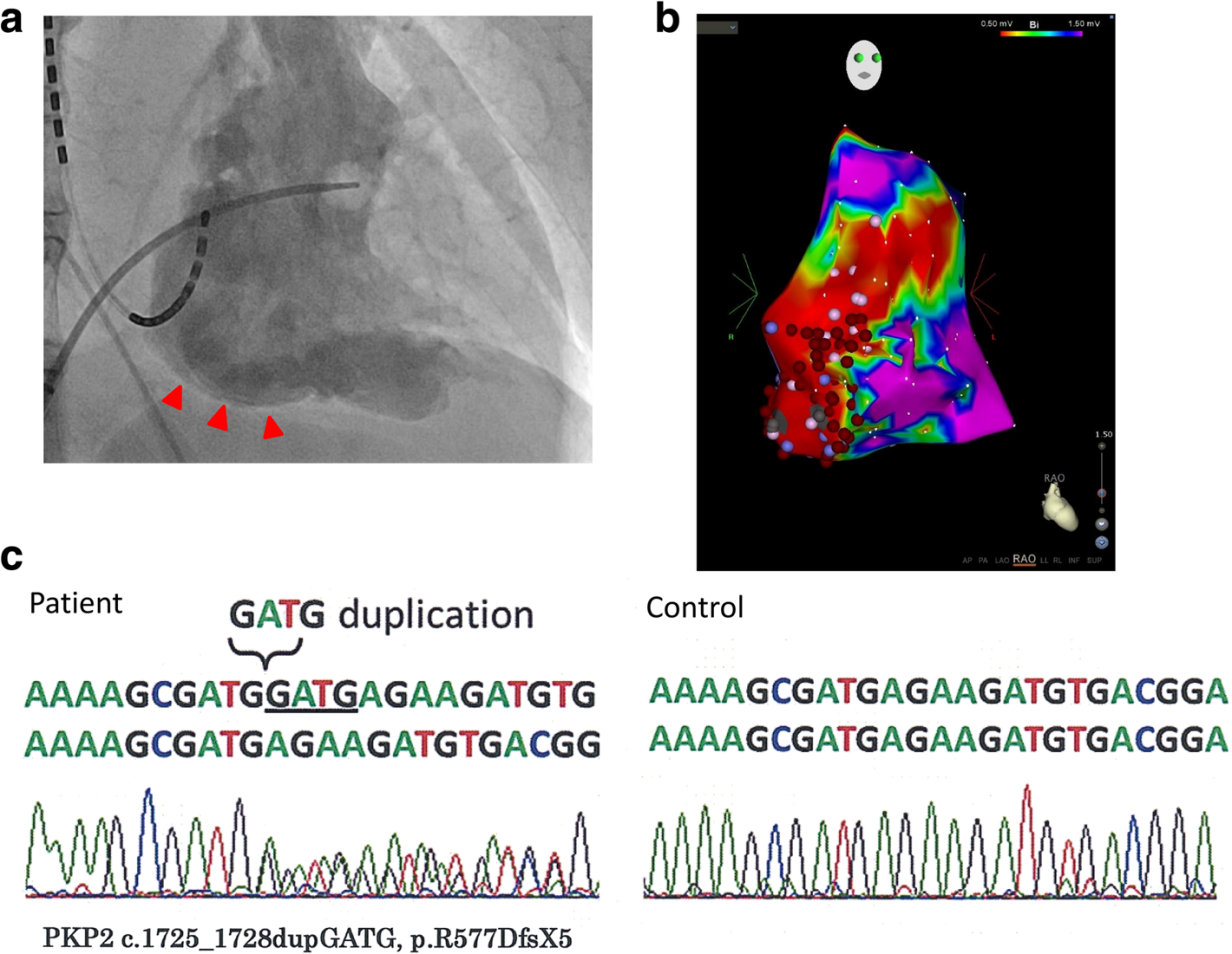
**a** Right anterior oblique (RAO) view of right ventriculography showing a right ventricular aneurysm (arrowheads). **b** RAO view of a right ventricular bipolar voltage map. Red areas represent dense scarring (amplitude < 0.5 mV). Areas with a color gradient between red and purple represent the border zone (amplitude of 0.5–1.5 mV). **c** Alignment of cDNA in the vicinity of codon 1725. The insertion mutation causes a frameshift and premature termination of translation (R577DfsX5)


An 80-year-old Japanese man with a history of hypertension presented to the emergency department for left shoulder pain. Although he was alert and conscious, his systolic blood pressure was 80 mmHg and his pulse was 204 beats/minute. A 12-lead electrocardiogram (ECG; Fig. 1a) showed ventricular tachycardia (VT). Electrical cardioversion was required because intravenous amiodarone failed to terminate the VT. His baseline ECG 1 month prior to this admission (Fig. 1b), which was recorded during hospitalization for appendicitis in another center, showed negative T waves in V1–V4 (Fig. 1b). Two-dimensional echocardiography showed normal left ventricular wall motion but severely reduced wall motion throughout the right ventricle. The right ventricular outflow tract was dilated on both the parasternal long (33 mm) and short (40 mm) axes and the right ventricular fractional area change was 26.2%. Coronary angiography was normal, but right ventriculography showed a right ventricular aneurysm (Fig. 2a, Additional file 1: Movie 1). The patient had no history of recent endurance exercise or participation in sports. Furthermore, a detailed family history revealed no cases of ARVC or sudden cardiac death. Although the patient didn’t give consent to endomyocardial biopsy, cardiac magnetic resonance imaging suggested diffuse areas of fat tissue in the right ventricular wall and also revealed late gadolinium enhancement in both right and left ventricular walls (Additional file 2).

**Additional file 1:** Movie 1. RAO view of right ventriculography showing a right ventricular aneurysm. (AVI 17715 kb)


Because this case met three major criteria (right ventricular aneurysm, inverted T waves in right precordial leads, and VT of left bundle-branch morphology with a superior axis), a clinical diagnosis of definite ARVC was established [3]. Regarding differential diagnosis, we considered cardiac sarcoidosis which can mimic ARVC. Although ^18^F fluorodeoxyglucose (FDG) positron emission scanning can be positive in some ARVC cases [4], cardiac sarcoidosis was deemed unlikely because abnormal FDG uptake was not observed in this patient (Additional file 3).

We recommended an implantable cardioverter-defibrillator; however, the patient rejected this suggestion and underwent an electrophysiological study and catheter ablation. A three-dimensional electroanatomical voltage map (Carto, Biosense-Webster Inc., CA, USA; Fig. 2b) during sinus rhythm showed an extensive low voltage zone in the right ventricle. Linear ablation along the low voltage zones with the pace mapping technique was performed. We then continued oral administration of amiodarone. The patient has been followed up for 3 years without recurrence of VT.

Genetic analysis identified a GATG duplication in exon 8 of *PKP2* (1725_1728dupGATG), which causes a frameshift and subsequent premature termination of translation (R577DfsX5) (Fig. 2c). The variant was not identified in the patient’s two daughters (51 and 49 years old) and is unknown in his parents because they are both deceased. Although this pathogenic variant is not reported in the genome aggregation database (gnomAD), previous reports have described this variant in Japanese ARVC patients [5, 6].

## Discussion

Current Task Force Criteria for an ARVC diagnosis combine diagnostic criteria from six categories including 1) global or regional dysfunction and structural alterations, 2) tissue characterization of the wall, 3) repolarization abnormalities, 4) depolarization/conduction abnormalities, 5) arrhythmias, and 6) family history [3]. This case met four major criteria so a diagnosis of definite ARVC was established.

Gerull and colleagues previously reported that pathogenic variants in *PKP2*, encoding the desmosomal protein plakophilin 2, are associated with ARVC [7]. Desmosomes are multiprotein structures of the cell membrane, which provide strong adhesion between cells. Armadillo-repeat proteins, as well as desmosomal cadherins and plakins, are known as structural constituents of desmosomes. Plakophilin 2 is a desmosomal armadillo-repeat protein, linking desmosomal cadherins with desmoplakin and the intermediate filament system [7]. Pathogenic variants of this gene are present in one-quarter of definite Japanese ARVC [6] and nearly half of Western European ARVC patients [8, 9].

A recent study of 502 ARVC patients revealed that 104 (21%) had a late presentation (age ≥ 50 years at diagnosis) and 3% were ≥ 65 years at diagnosis with a mean age of 71 years (range, 65–76 years) [10]. Interestingly, late presentation of symptoms did not necessarily indicate a low risk for disease-related morbidity and mortality, but showed a similar arrhythmic time course as that for earlier onset symptoms. Late presentation was reportedly associated with male sex, pathogenic variant, right ventricular structural disease, lack of family history, and electrophysiological study inducibility [10]. The present case shared four of these characteristics (male sex, pathogenic variant, right ventricular structural disease, and lack of family history). Limitations of our report include the possibility that ARVC was missed on prior examination. Nevertheless, a detailed interview revealed no previous arrhythmia history. We also requested ECG reports from local hospitals including information from medical checkups; however, none were obtained. Regardless of this, our findings reveal important characteristics of ARVC with late presentation.

## Conclusions

To the best of our knowledge, this is the first report of newly diagnosed ARVC with a *PKP2* pathogenic variant in an octogenarian. Although the late clinical presentation of ARVC is rare, it should be included in the differential diagnosis when treating older patients with ventricular tachyarrhythmias.

## Additional file

Additional file 2: Cardiac magnetic resonance imaging. a. T1-weighted black-blood (T1BB) imaging of a short axis view indicating diffuse areas of fat tissue in the right ventricular wall (yellow arrows). b. T1BB imaging with fat saturation (fat-sat) of a short axis view at the same level as the T1BB imaging (panel a). c. Late gadolinium enhancement (LGE) in the right ventricular wall in a short axis view (red arrows). d. Late gadolinium enhancement in the mid-wall of the interventricular septum in a short axis view (red arrows). (TIFF 5156 kb)

Additional file 3: ^18^F fluorodeoxyglucose (FDG) positron emission tomography (PET) with computed tomography (CT) a A transverse view of FDG-PET/CT. b. A coronal view of FDG-PET/CT. Abnormal FDG uptake was not detected in left ventricle (LV) or right ventricle (RV). (TIFF 4263 kb)

## Abbreviations

ARVC: Arrhythmogenic right ventricular cardiomyopathy
cDNA: Complementary DNA
ECG: Electrocardiogram
FDG: ^18^F fluorodeoxyglucose
gnomAD: Genome aggregation database
LGE: Late gadolinium enhancement
LV: Left ventricle
PKP2: Plakophilin 2
RAO: Right anterior oblique
RV: Right ventricle
T1BB: T1-weighted black-blood
VT: Ventricular tachycardia
