# Supplementary figures and images for: Late presentation of arrhythmogenic right ventricular cardiomyopathy in an octogenarian associated with a pathogenic variant in the plakophilin 2 gene: a case report
Source: BMC Cardiovasc Disord. 2019 Feb 19;19:41. doi: 10.1186/s12872-019-1018-2 (PMC6381698; doi:10.1186/s12872-019-1018-2)

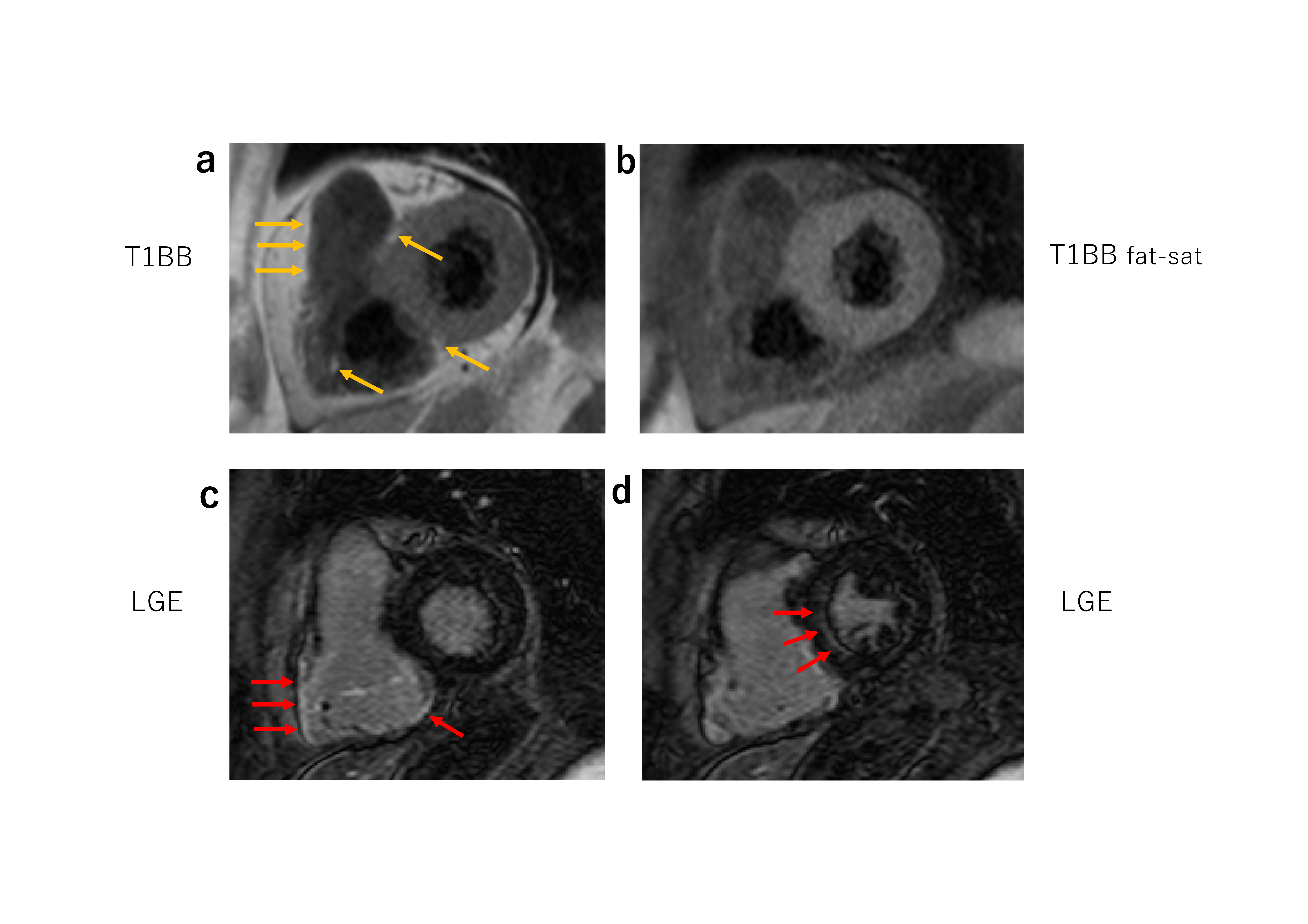

Supplement: Supplementary file 2 — Cardiac magnetic resonance imaging. a. T1-weighted black-blood (T1BB) imaging of a short axis view indicating diffuse areas of fat tissue in the right ventricular wall (yellow arrows). b. T1BB imaging with fat saturation (fat-sat) of a short axis view at the same level as the T1BB imaging (panel a). c. Late gadolinium enhancement (LGE) in the right ventricular wall in a short axis view (red arrows). d. Late gadolinium enhancement in the mid-wall of the interventricular septum in a short axis view (red arrows). (TIFF 5156 kb) [file 12872_2019_1018_MOESM2_ESM.tiff]

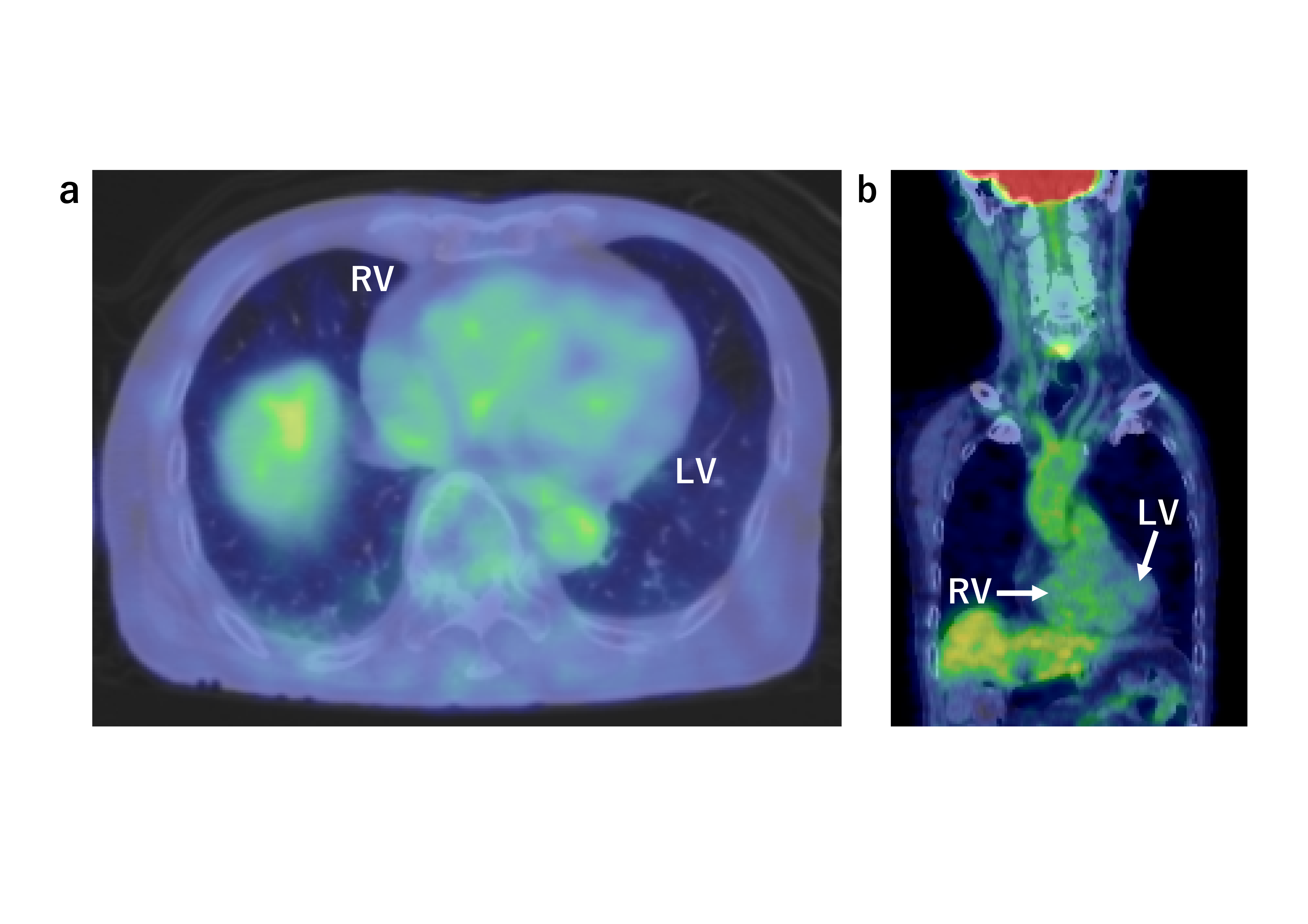

Supplement: Supplementary file 3 — 18F fluorodeoxyglucose (FDG) positron emission tomography (PET) with computed tomography (CT) a A transverse view of FDG-PET/CT. b. A coronal view of FDG-PET/CT. Abnormal FDG uptake was not detected in left ventricle (LV) or right ventricle (RV). (TIFF 4263 kb) [file 12872_2019_1018_MOESM3_ESM.tiff]
